# Un mode de révélation rare du kyste hydatique hépatique: la rupture intrapéritonéale, à propos de 5 cas

**Published:** 2011-03-18

**Authors:** Mohamed Rami, Khalid Khattala, Abdelhalim Mahmoudi, Aziz EL Madi, My Afifi Abderrahmane, Youssef Bouabdallah

**Affiliations:** 1 Service de chirurgie pédiatrique, CHU Hassan II, Fès, Maroc

**Keywords:** Kyste hydatique, rupture, peritoine, hépatique, Maroc

## Abstract

Le kyste hydatique est une pathologie fréquente dans notre pays, toutes les localisations sont possibles. La révélation par un abdomen aigu est rare, nous rapportons 5 cas colligés au service de chirurgie pédiatrique de Fès de février 2004 à août 2008. Il s’agit de 5 enfants, avec une moyenne d’âge de 12 ans, victimes d’une contusion abdominale suite à une chute avec impact au niveau de l’hypochondre droit. Ils ont été reçus aux urgences dans un tableau d’abdomen aigu. L’imagerie objective des kystes rompus, avec une hydatidose hépatico-péritonéale dans un cas, une double localisation hépatico-rénale dans un cas. Ils ont bénéficié d’une cure chirurgicale, avec un traitement médical antiparasitaire associé en postopératoire pendant six mois. Deux patients ont développé un rush cutané avec urticaire, qui a disparu sous traitement symptomatique. L’évolution est bonne chez tous les patients avec un recul moyen de 4 ans et demi.

## Introduction

L’hydatidose hépatique est fréquente dans nos contrées. La rupture dans la cavité péritonéale est un accident rare réalisant des tableaux cliniques polymorphes. L’échographie et le scanner abdominal permettent le plus souvent un diagnostic facile et précis de cette complication. Nous rapportons le cas de 5 patients pris en charge au service de chirurgie du CHU Hassan II de Fès (Maroc) chez qui la rupture intrapéritonéale a été le mode de révélation de kyste hydatique hépatique

## Patients et observations

**Observation 1**

Il s’agit d’un garçon de 10 ans, victime d’une chute sur l’hypochondre droit, entraînant un abdomen aigu, avec vomissements. L’examen trouve un patient fébrile, une défense de l’hypochondre droit, sans masse palpable. Le bilan biologique trouve une hyperleucocytose à 15 000 E/mm^3^. L’échographie abdominale objective un kyste hydatique type II, avec un épanchement intrapéritonéal. Le patient a été mis sous surveillance, avec remplissage et antibiothérapie. Devant la persistance des symptômes avec apparition d’une réaction allergique, le patient a été opéré. Nous avons découvert un kyste rompu, avec des vésicules filles en intrapéritonéal. Nous avons réalisé une toilette au sérum salé et sérum hypertonique, avec résection du dôme saillant. Le patient est resté sous antibiothérapie, corticothérapie, et traitement médical à base d’albendazole pendant 6 mois (cure de 15 jours par mois). Le recul est de 6 ans.

**Observation 2**

Il s’agit d’une fille de 11 ans, victime d’une contusion abdominale deux jours auparavant. L’examen initial trouve une patiente fébrile, avec une sensibilité généralisée et une matité des flancs. Le bilan biologique montre une hyperleucocytose à 14 300 E/mm^3^. L’échographie faite en urgence, objective une hydatidose hépatique, avec un kyste rompu, ainsi qu’un épanchement intrapéritonéal. La patiente a été opérée aux urgences, mise sous antibiothérapie et traitement antiparasitaire (pendant 6 mois), avec bonne évolution. Le recul est de 6 ans.

**Observation 3**

Il s’agit d’un garçon de 12 ans, sans antécédents, victime d’une chute de sa hauteur avec impact au niveau de l’hypochondre droit. L’examen trouve un patient apyrétique, bon état hémodynamique, avec une sensibilité abdominale généralisée et un maximum au niveau de l’hypochondre droit. Le bilan biologique objective une hyperleucocytose à 11 200 E/mm^3^, et une hyperéosinophilie à 2839 E/mm^3^. L’échographie montre un épanchement intrapéritonéal de grande abondance, avec un kyste hydatique type II de douze cm, rompu. La TDM abdominale confirme les données de l’échographie. Le patient est mis sous triple antibiothérapie, et a bénéficié d’une cure chirurgicale. Les suites opératoires sont simples, le patient a été mis sous cure d’albendazole pendant 6 mois, qu’il n’a suivi que pendant trois mois, avec bonne évolution. Le recul est de 4 ans et demi.

**Observation 4**

**Figure 1:**
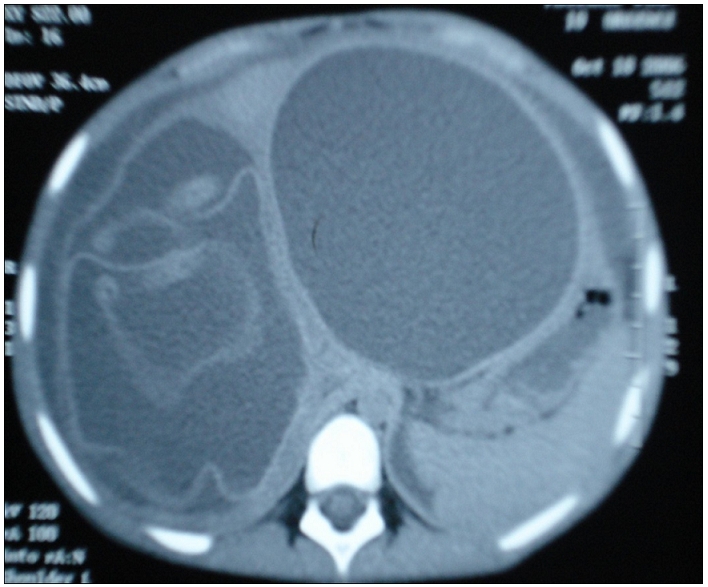
Aspect scannographique d’un kyste hépatique rompu

Il s’agit d’une fille de 11 ans, traitée pour une tuberculose pulmonaire à l’âge de trois ans, opérée pour un kyste hydatique hépatique à l’âge de 9 ans. Admise aux urgences pour un abdomen aigu suite à une contusion abdominale. L’examen trouve une patiente fébrile, avec douleur abdominale, et vomissements. La palpation objective une masse hépatique ferme, douloureuse, avec matité des flancs. Le bilan biologique trouve une hyperleucocytose à 13 000 E/mm^3^. L’échographie et le scanner abdominal ont révélé deux kystes hydatiques hépatiques dont l’un était rompu, et un kyste rénal type III (Figure 1 et 2). La patiente a été opérée en urgence pour son kyste hépatique rompu. Elle a développé un rush cutané avec épisode d’hypotension, qui a bien évolué sous corticothérapie et traitement antiparasitaire. La patiente a bénéficié deux mois plus tard de la cure de son kyste rénal. La patiente a été mise sous traitement médical à base d’albendazole pendant 6 mois. Le recul est de 4 ans.

**Observation 5**

**Figure 3:**
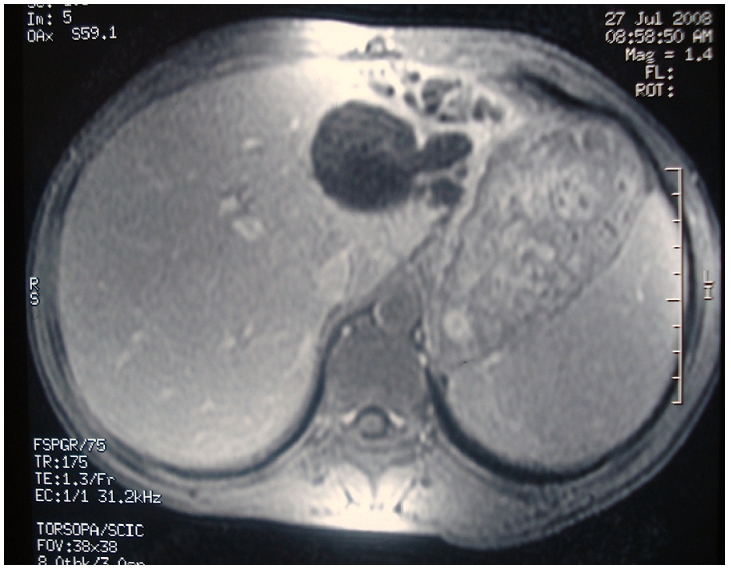
IRM montrant une hydatidose hépatique avec atrophie du foie gauche

Il s’agit d’un garçon de 15 ans, victime d’une chute de sa hauteur. Le patient a présenté une douleur de l’hypochondre droit accompagnée d’un syndrome fébrile. A l’examen, on trouvait un tableau d’angiocholite, fait d’une fièvre à 38°8, un ictère cutanéo-muqueux, et une défense de l’hypochondre droit, signe de Murphy positif. Le bilan biologique montrait une hyperleucocytose à 16800 E/mm^3^. L’échographie a montré une hydatidose hépatique, avec une dilatation des voies biliaires. Un complément par une bili-IRM a confirmé ces données, et permis de montrer une atrophie du foie gauche (Figure 3 et 4) avec des membranes au sein de la voie biliaire principale, expliquant sa dilatation. Après réanimation du patient et l’instauration d’une antibiothérapie, la cure chirurgicale a consisté en : toilette péritonéale soigneuse, résection du dôme saillant, cholécystectomie et lavage de la voie biliaire principale au sérum salé hypertonique. Le patient a été mis sous traitement médical à base d’albendazole pendant 6 mois.

## Discussion

Du point de vue du cycle parasitaire, le ver adulte est un parasite du chien. Les herbivores, et en particulier le mouton, sont des hôtes intermédiaires. La contamination se fait par voie digestive. L’embryon d’E. granulosus gagne le système porte, le foie puis tous les organes par voie systémique. L’hydatide ainsi constituée peut atteindre une grande taille, se rompre, essaimer et vésiculer à distance, réalisant l’échinococcose secondaire. Au niveau géographique, il existe quatre foyers principaux d’hydatidose: le bassin méditerranéen, l’Amérique du sud, l’Australie, et

l’Asie centrale Le sud-est de la France est un foyer très actif. La localisation préférentielle chez l’enfant est pulmonaire (50%) puis hépatique (40%).Au cours de l’hydatidose hépatique, il n’existe initialement qu’une hépatomégalie isolée, homogène, indolore ou sensible [1-3].

La rupture du kyste hydatique du foie dans la cavité péritonéale est une complication rare, sa fréquence est estimée dans la littérature entre 1,7% et 7%. Cette rupture est aiguë dans 20,5% des cas, chronique réalisant une échinococcose péritonéale secondaire dans 63,5% des cas.

L’analyse des ruptures intra-péritonéales permet de distinguer deux formes cliniques : les fissurations minimes et les ruptures massives. Les fissurations minimes sont les plus fréquentes, et résultent d’un traumatisme qui est le plus souvent méconnu ou négligé. L’interrogatoire retrouve dans quelques cas la notion d’une augmentation progressive du volume de l’abdomen après affaissement d’une masse connue, associée ou non à une éruption cutanée transitoire. Ainsi se déversent en faible quantité dans la grande cavité péritonéale du liquide hydatique, des vésicules et des scolex qui peuvent, soit s’enkyster réalisant une échinococcose péritonéale vésiculeuse enkystée, soit resté libres, réalisant une véritable miliaire hydatique. Les ruptures massives lors d’un grand effort sont beaucoup plus rares. Elles entraînent l’affaissement rapide du kyste par vidange complète dans la cavité péritonéale. Elles peuvent évoluer à bas bruit réalisant une forme subaiguë ou de façon bruyante réalisant une forme aiguë [1-5].

Le tableau clinique est fait d’un syndrome douloureux aigu de l’épigastre et/ou de l’hypochondre droit accompagné de vomissements et d’un état de choc plus ou moins marqué, avec à l’examen clinique un syndrome péritonéal franc. Des manifestations allergiques peuvent s’associer allant de l’accident dramatique de choc anaphylactique avec œdème de Quick, et de manifestations plus atténuées à type de prurit, d’urticaire, de dyspnée asthmatiforme ou d’un malaise vagal de grande valeur diagnostique [6]. Ailleurs, la rupture est peu symptomatique et de découverte opératoire dans environ 5,6% des cas.

L’infection de la cavité péritonéale peut se faire de deux manières : soit d’emblée par la rupture d’un kyste hydatique initialement infecté, soit plus tardivement par surinfection.

Sur le plan biologique, les fonctions hépatiques sont le plus souvent normales et l’hyperéosinophilie absente, sauf après fissuration ou rupture du kyste. Le diagnostic est donc sérologique par association d’une technique qualitative et d’une quantitative. Les techniques qualitatives comprennent l’électrosynérèse, le Western-Blot et l’immunoélectrophorèse.

L’échographie est d’un grand apport diagnostic. Elle permet d’objectiver le kyste en montrant son siège, de suspecter la rupture sur certains signes indirects : l’affaissement partiel ou total de la cavité kystique et la présence d’un épanchement péritonéal échogène. La tomodensitométrie vient confirmer les données échographiques en montrant une formation liquidienne hétérogène contenant une structure dense serpigineuse correspondant au kyste rompu et un épanchement intra-péritonéal [7].

Le traitement chirurgical de cette complication doit obéir à certains principes: Une toilette abondante au sérum physiologique additionné de solution scoliocides dans le but d’éviter les récidives péritonéales (sérum salé hypertonique à une concentration de 33% serait le plus efficace et le moins nocif).

Le traitement du kyste : la résection du dôme saillant, est la technique la plus utilisée dans le contexte de l’urgence (83%) [8].

Un traitement médical adjuvant semble nécessaire, à base d’albendazole, sous forme de cures de 15 jours par mois pendant 6 mois, avec contrôle de la fonction hépatique. Lors des manifestations allergiques, et selon la gravité, on prescrira des antihistaminiques voire une corticothérapie en bolus en cas de choc anaphylactique [9,10].

Dans l’immédiat, le pronostic est lié à la gravité de la péritonite. La mortalité dans les séries récentes est faible de l’ordre de 2,9% comparée aux séries anciennes où la mortalité peut atteindre 43 à 50%. La morbidité est liée non pas à la péritonite mais aux complications spécifiques du kyste: la fistule biliaire externe et la suppuration de la cavité résiduelle. Ultérieurement, c’est le problème de l’échinococcose péritonéale secondaire observée dans environ 63,5% des cas.

## Conclusion

La rupture de kyste hydatique du foie dans la cavité péritonéale est un accident rare réalisant des tableaux cliniques polymorphes. L’examen tomodensitométrique et l’échographie permettent le plus souvent un diagnostic facile et précis de cette complication. Le traitement est chirurgical, encadré par un traitement médical. Le suivi échographique à distance du geste chirurgical est capital afin de dépister les récidives et les localisations secondaires.

## Contribution des auteurs

Tous les auteurs ont contribué lors de la réflexion diagnostique, ainsi que pour le traitement et le suivi des patients et la rédaction du manuscrit.

## Figures and Tables

**Figure 2:**
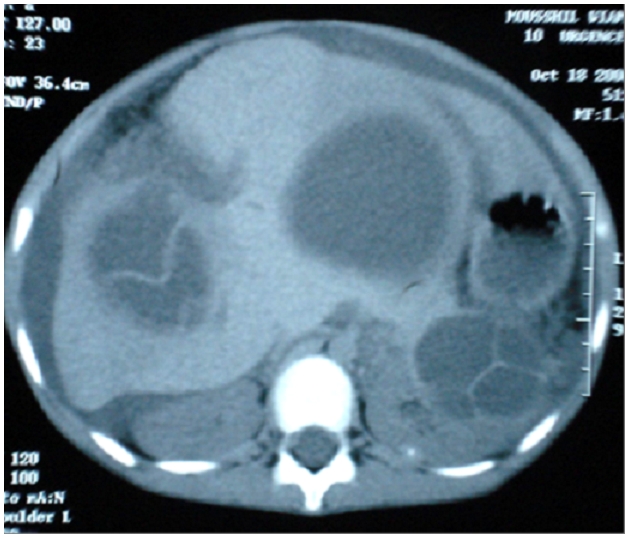
Double localisation hépatico-rénale, avec rupture du kyste hépatique, épanchement péritonéal

**Figure 4:**
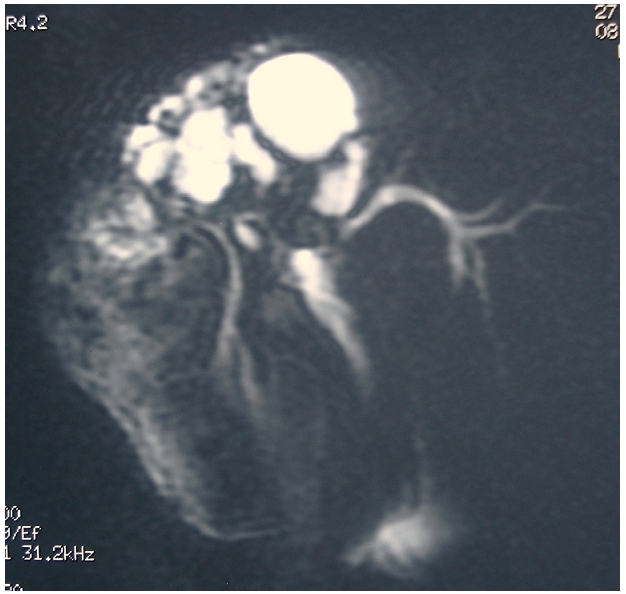
Bili-IRM montrant plusieurs kyste, avec rupture dans les voies biliaires dilatées
